# Action needed for the EU Common Agricultural Policy to address sustainability challenges

**DOI:** 10.1002/pan3.10080

**Published:** 2020-03-08

**Authors:** Guy Pe'er, Aletta Bonn, Helge Bruelheide, Petra Dieker, Nico Eisenhauer, Peter H. Feindt, Gregor Hagedorn, Bernd Hansjürgens, Irina Herzon, Ângela Lomba, Elisabeth Marquard, Francisco Moreira, Heike Nitsch, Rainer Oppermann, Andrea Perino, Norbert Röder, Christian Schleyer, Stefan Schindler, Christine Wolf, Yves Zinngrebe, Sebastian Lakner

**Affiliations:** 1 German Centre for Integrative Biodiversity Research (iDiv) Halle-Jena-Leipzig, Leipzig, Germany; 2 Helmholtz Centre for Environmental Research - UFZ, Leipzig, Germany; 3 Leipzig University, Leipzig, Germany; 4 Institute of Biodiversity, Friedrich Schiller University Jena, Jena, Germany; 5 Institute of Biology/Geobotany and Botanical Garden, Martin Luther University Halle-Wittenberg, Halle/S., Germany; 6 Thünen Institute of Biodiversity, Braunschweig, Germany; 7 Thaer Institute for Agricultural and Horticultural Sciences, Agricultural and Food Policy Group, Humboldt-Universität zu Berlin, Berlin, Germany; 8 Scientists for Future, Berlin, Germany; 9 Department of Agricultural Sciences and Helsinki Institute of Sustainability Science, University of Helsinki, HELSUS, Helsinki, Finland; 10 CIBIO-InBIO, University of Porto, Vairao, Portugal; 11 Institute of Agronomy, CIBIO-InBIO, University of Lisbon, Lisbon, Portugal; 12 Institute for Rural Development Research, Frankfurt-am-Main, Germany; 13 Institute for Agroecology and Biodiversity (IFAB), Mannheim, Germany; 14 Thünen Institute for Rural Studies, Braunschweig, Germany; 15 Institute of Geography, University of Innsbruck, Innsbruck, Austria; 16 Division of Conservation Biology, Vegetation and Landscape Ecology, University of Vienna, Vienna, Austria; 17 Faculty of Environmental Sciences, Community Ecology and Conservation Research Group, Czech University of Life Sciences Prague, Prague 6, Czech Republic; 18 Department for Agricultural Economics and Rural Development, Georg-August-Universität Göttingen, Göttingen, Germany

**Keywords:** agriculture, biodiversity, climate change, Common Agricultural Policy, European Green Deal, evidence-based policy, public goods, SMART targets

## Abstract

1. Making agriculture sustainable is a global challenge. In the European Union (EU), the Common Agricultural Policy (CAP) is failing with respect to biodiversity, climate, soil, land degradation as well as socio-economic challenges.
2. The European Commission's proposal for a CAP post-2020 provides a scope for enhanced sustainability. However, it also allows Member States to choose low-ambition implementation pathways. It therefore remains essential to address citizens' demands for sustainable agriculture and rectify systemic weaknesses in the CAP, using the full breadth of available scientific evidence and knowledge.
3. Concerned about current attempts to dilute the environmental ambition of the future CAP, and the lack of concrete proposals for improving the CAP in the draft of the European Green Deal, we call on the European Parliament, Council and Commission to adopt 10 urgent action points for delivering sustainable food production, biodiversity conservation and climate mitigation.
4. Knowledge is available to help moving towards evidence-based, sustainable European agriculture that can benefit people, nature and their joint futures.
5. The statements made in this article have the broad support of the scientific community, as expressed by above 3,600 signatories to the preprint version of this manuscript. The list can be found here (https://doi.org/10.5281/zenodo.3685632).

## 1 Agriculture is the Main Driver of Environmental Degradation in Europe

**Figure 1:**
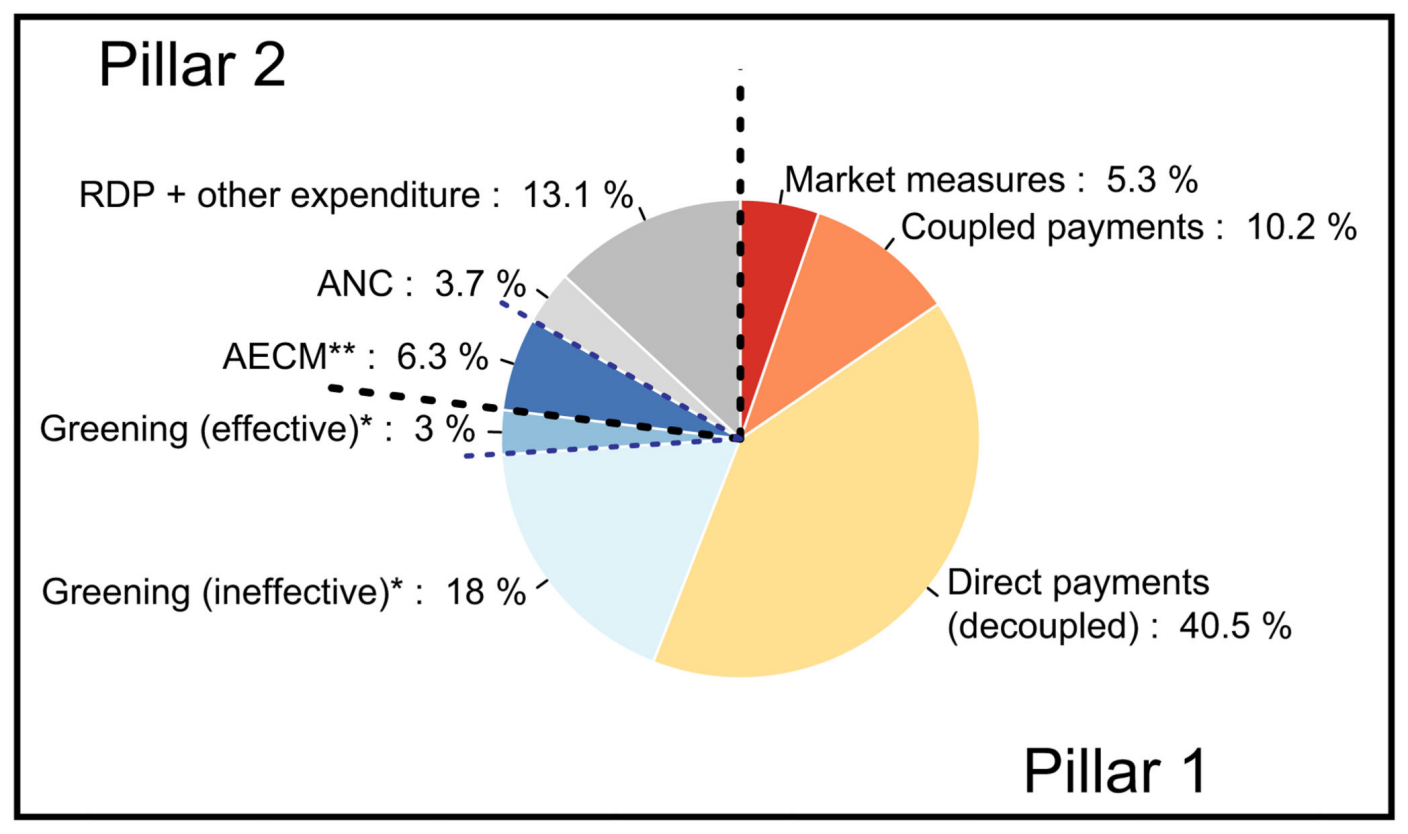
Common Agricultural Policy (CAP) expenditures for key instruments in 2019. *Source*: EC (2019c) for total budget-appropriations. *Notes*: (1) *Shares of effective versus ineffective Greening are based on Pe'er, Zinngrebe, et al. (2017). (2) **Shares of Agri-Environmental and Climate Measures (AECM), Areas of Natural Constraints (ANC) and Rural Development Programmes (RDP) are estimated based on EC (2016)

Agricultural expansion and intensification are key drivers of biodiversity and ecosystem services loss (Diaz et al., 2019) as well as climate change (IPCC, 2019). His torically some agricultural practices supported biodiversity and multiple ecosystem services. Yet such practices have been increasingly abandoned or replaced by farming systems which maximize yields through unsustainable use of natural resources and at the expense of biodiversity and ecosystem services (Stoate et al., 2009). These processes are driven by socio-economic and technological forces but also supported by public policies. The European Union's (EU) Common Agricultural Policy (CAP; see Box 1) shapes the EU's agricultural sector (Hodge, Hauck, & Bonn, 2015) and supports a variety of practices contributing to wide-scale biodiversity loss (Gregory, Skorpilova, Vorisek, & Butler, 2019; Pe'er et al., 2014, 2019; Van Swaay et al., 2019), climate change (Alliance Environment, 2019), soil erosion (Orgiazzi et al., 2016) and land degradation (IPBES, 2018). CAP programmes that could counteract these developments have been insufficient and/or underfunded (Alliance Environment, 2019; Pe'er et al., 2019). Furthermore, the current CAP is ineffective and inefficient also in addressing the social and economic challenges (ECA, 2016; Pe'er, Lakner, et al., 2017). The CAP has undergone several reforms, partly aiming to enhance its environmental and social performance. Some positive outcomes have been described (Batáry, Dicks, Kleijn, & Sutherland, 2015; Walker et al., 2018), yet effective measures are required to reverse negative trends (e.g. Pe'er, Zinngrebe, et al., 2017)—indicating the need of a much more fundamental change of the CAP to deliver on both environmental and socio-economic challenges. The CAP post-2020, as proposed by the European Commission in June 2018 (EC, 2018), acknowledges the need to address environmental and sustainability challenges and introduces a new Green Architecture and a delivery model that offers Member States (MSs) higher flexibility as to how they implement the CAP (see Box 1; Figure 1). The post-2020 CAP proposal is currently under final phases of negotiations.

## 2 Reasons for Concern

Analysis of the Commission's proposal indicates that it generally retains the structure and weaknesses of the current CAP (see also Pe'er et al., 2019). Key shortcomings include:

- **Continuation of subsidies through area-based ‘Direct Payments’ (in Pillar 1) with low levels of environmental requirements.** Area-based payments are inefficient both with respect to farmers' income and environmental aims, and their recent ‘greening’ has achieved minimal change in agricultural practice and environmental performance – only <5% of the area under greening has seen a change in management (ECA, 2017). Direct Payments are often passed on to landowners rather than benefiting those who manage the land (Hennig & Breustedt, 2018; WBAE, 2018), and attempts to cap the maximum allowable payments, and redistribute the funds to address their highly inequitable distribution, are likely to remain unsuccessful (Matthews 2018a, 2018b). Moreover, the coupling of some Direct Payments to high-input production remains permitted despite strong evidence that Coupled Payments lead to market distortion (OECD, 2017), foster greenhouse gas (GHG) emissions and support practices with demonstrated negative impacts on biodiversity (Pe'er, Lakner, et al., 2017).
- **Budget cuts for Rural Development Programmes (so-called Pillar 2), including Agri-Environment-Climate Measures (AECM).** If designed and implemented well, these policy tools are the most effective in supporting pro-environmental farming practices (Batáry et al., 2015; Walker et al., 2018). Cutting the respective budgets, rather than reducing barriers to effective implementation (such as insufficient funding, high administrative complexity and insufficient incentives for uptake), are therefore counterproductive (CEJA et al., 2019).
- **Misleading claims attached to insufficient climate action.** The Commission's proposal states that 40% of the expenditures for Direct Payments and Support for Areas of Natural Constraints (ANC) will be labelled as ‘climate-friendly’. Yet these instruments are not systematically linked to any effective measure for greenhouse gas reduction or climate adaptation, thus lacking any justification of this statement. Instead, they even partly support practices and sectors with significant greenhouse gas emissions (Alliance Environment, 2019; Pe'er et al., 2019).
- **A ‘Green Architecture’ with vague requirements allows MSs and farmers to choose unambitious (‘light green’) options**. The Commission's proposal presents a new voluntary instrument (‘Eco-Schemes’) and a slightly expanded set of environmental conditions under ‘Cross Compliance’ for Direct Payments. The proposal also demands higher ambition from the MSs on the environmental performance compared to the current period (article 92 in EC, 2018). However, the proposal fails to list concrete measures that are known as essential for biodiversity and environment and thus should be prioritized by MSs, such as maintaining and restoring small-scale landscape features (see Harvey et al., 2020), buffer strips, fallow land, high-diversity grasslands, and at the landscape level, viability of High Nature Value farmland regions (Navarro & López-Bao, 2019). While flexibility for MSs and farmers to make their own choices is valuable for developing context-specific solutions, the experience of past and current CAP cycles is that a lack of clear requirements and evaluation criteria encourages a ‘race to the bottom’ where MSs ‘compete’ for the lowest requirements for their farmers’ Direct Payments (Heinemann & Weiss, 2018; WBAE, 2018). The proposed ‘performance bonus’ (article 123 in EC, 2018), which should incentivize MSs to meet their goals, may adversely fuel such a race by incentivizing MSs to set easy-to-achieve targets from the onset.
- **Insufficient set of indicators** (Annex I of EC, 2018). The planned ‘output’ and ‘results’ indicators basically monitor the administrative and financial implementation of the CAP. The proposed ‘impact’ indicators mostly describe farming structures rather than actual impacts. They are insufficient for an effective monitoring of the CAP objectives and instruments and provide little guidance for policy steering. For example, there is a lack of indicators on farm management, land-use and land cover, environmental parameters and the economic performance of farming households (Pe'er et al., 2019; WBAE, 2018). This stands in stark contradiction to the result-based principles that the future CAP is proposed to follow. Moreover, complex administrative burdens that are disproportionate to their simplistic contents, set major hurdles to ambitious environmental implementation by MSs (WBAE, 2019a, 2019b).
- **Extending insurance instruments without a link to risk mitigation can promote unsustainable, risk-prone behaviour**. Extending risk management tools (i.e. insurance; article 70 in EC, 2018) seems reasonable given the increased risks to farmers from market exposure, environmental degradation (partly due to overuse of resources) and climate change (especially extreme weather events such as heat, droughts and wildfires). Climate change also enhances sanitary hazards (through pests and pathogens) and phytosanitary hazards (through plant pathogens; Altizer, Ostfeld, Johnson, Kutz, & Harvell, 2013; Velásquez, Castroverde, & He, 2018). However, without requiring proper risk mitigation measures, insurance may promote risk-prone behaviour, that is, disregarding avoidable risks (Goodrich, Yu, & Vandeveer, 2020; Müller, Johnson, & Kreuer, 2017).
- **Lack of consistency and transparency**. The proposed CAP post-2020 repeats the heavily criticized procedure of restructuring and renaming CAP elements in a way that impedes learning and undermines transparency and legitimacy (Erjavec & Erjavec, 2015; Rutz, Dwyer, & Schramek, 2014), rather than conducting a deep reform. Previous reforms have failed to redesign or integrate existing instruments to improve the CAP's performance (Alons, 2017; Feindt, 2010; Pe'er et al., 2014, 2019; Simoncini et al., 2019). Along the same line, the Commission's proposal for the CAP post-2020 retains vagueness in its guidance for implementation, thereby risking a dilution of ambition in implementation. On top of that, there are current pressures to water down further the environmental requirements set by the CAP. This can be evidenced in the amendments voted for by the European Parliament's Committee for Agriculture and Rural Development (COMAGRI, 2019), and in a draft proposal from the EU's Council (representing the MSs) which reduces or removes a range of environmental requirements (Council of the European Union, 2019). Both of these show that, as in the previous reform cycle, a closed institutional process is used to defend the interests of a few at the expense of many (Erjavec & Erjavec, 2015)—thereby, disregarding both public calls for decisive action on the environment and the robust scientific evidence indicating the need for a profound policy change (Matthews, 2017; Pe'er, Lakner, et al., 2017).

The ‘European Green Deal’, published by the European Commission in December 2019, presents a new framework for EU policy-making with high ambition to align economic processes with planetary boundaries. It states an intention to present a ‘Farm to Fork Strategy on sustainable food’ (EC, 2019b; von der Leyen, 2019). This may offer an important opportunity for the European Institutions to make evidence-based decisions toward a future-proof CAP. However, the Green Deal is vague with respect to the CAP. It reiterates that ‘at least 40% of the Common Agricultural Policy's overall budget […] would contribute to climate action’ (EC, 2019b, p. 12), a claim already assessed as unjustifiable (EC, 2019b; Pe'er et al., 2019). Beyond a focus on the Strategic Plans required by the MSs for implementation, there is little indication on how the Commission intends to address the systemic flaws of the CAP, and the shortcomings outlined above.

## 3 Ten Action Points

We call on the European Commission, Parliament, the Council and MSs to use the breadth of scientific knowledge and experiences from past CAP reforms for drastically improving the CAP in order to avoid a policy failure and further ineffective use of taxpayers' money. **As an overarching target, all CAP elements, without exception, should be aligned with the principles of sustainability, multifunctionality and public payments for public goods**. We propose 10 urgent action points, accompanied by targets and implementation options (Table 1), to focus on 40% of the EU budget on public goods and societal objectives and improve the management of half of the EU's land area.

1. **Transform Direct Payments into payments for public goods**, to align both environmental and socio-environmental dimensions of sustainability, given the poor performance of Direct Payments for both (Navarro & López-Bao, 2019). Most urgent would be the abolishment of Coupled Payments for intensive production systems with high GHG emissions but low delivery of public goods, and to diminish the distortion of markets (OECD, 2017). Transforming Direct Payments would allow using public funds in a more target-oriented way, be it as funds for Eco-Schemes, for gradual expansion of Rural Development, to improve support for multi-functional farming systems that are designed according to agroecological principles like organic farming and agroforestry (Lampkin et al., 2015), or to help protect High Nature Value farming systems (EIP-AGRI Focus Group, 2016).
2. **Provide sufficient support for effective climate change mitigation**, aiming to reduce GHG emissions in the agricultural sector with a focus on improved nitrogen fertilizer application, rewetting of peatlands and improved GHG balances from livestock husbandry (WBAE, 2016). Insurance against climate-related risks should be conditional on tangible risk mitigation measures for droughts, wildfires, floods, soil losses and GHG emissions, for example through relevant landscape features and proper management of vegetation and soil cover.
3. **Provide sufficient support for effective instruments to maintain biodiversity and ecosystems**, aiming to halt and reverse ongoing declines in farmland biodiversity (Mace et al., 2018). This can be done by securing and enhancing budgets for AECM and Eco-Schemes and other environmental measures in both Pillars; restoring the pre-2009 requirements for Member States to set aside at least 10% of Utilized Agricultural Area for nature and semi-natural habitats; expanding support for low-input production without or with minimal chemical fertilizers or pesticides (e.g. organic farming), expansion and longer-term maintenance of fallow land (Pe'er, Zinngrebe, et al., 2017) and extensive grazing on High Nature Value farmland; channelling support to efficient (so-called ‘dark green’) measures; and achieving a coherent and synergistic policy design across Pillars (e.g. Lakner, Holst, Dittrich, Hoyer, & Pe'er, 2019).
4. **Promote innovative approaches to design and implement measures addressing the environmental challenges**, such as result-based remuneration of AECM (e.g. oriented to target species or habitats, Herzon et al., 2018), collective measures to support landscape-level management (see below) or the introduction of a points system to reward farmers for their ambition and/or investments, as also proposed by several farmer organizations (e.g. Neumann, Dierking, & Taube, 2017).
5. **Enhance spatial planning and collaborative implementation of landscape-level measures**, as such approaches have been shown to be successful with respect to environmental aims (Westerink et al., 2017). Policy ‘experiments’ are urgently needed, for both Pillars, to allow local targeting of management measures that can achieve a more effective delivery of public goods such as maintaining water quality (Jones et al., 2017; Lomba et al., 2020), reducing fire hazard (Moreira & Pe'er, 2018) and contributing to the EU's strategy on Green Infrastructure. Such approaches should entail longer-term contracts with farmers to improve income security and ecological benefits.
6. **Require MSs to set S.M.A.R.T. targets in their Strategic Plans** (i.e. specific, measurable, ambitious, realistic and time-bound; Green et al., 2019) in order to fulfil all CAP objectives. This is essential for aligning the CAP with other national and international policies and commitments. MSs should be obliged to demonstrate how they address trade-offs between objectives (see Supporting Information in: Pe'er et al., 2019). This will require guidance by the Commission, as well as close monitoring of implementation and outcomes.
7. **Revise the set of indicators** to ensure they are supported by the best available science and comply with the indicators of the Sustainable Development Goals (SDGs), the Convention for Biological Diversity (CBD) and the United Nations' Framework Convention on Climate Change (UNFCCC). Implementing a result-based approach requires both result and impact indicators to be adequate and meaningful (Herzon et al., 2018). For example, well-established biodiversity indicators such as the Butterfly Grassland Indicator (Van Swaay et al., 2019) should be added to complement the Farmland Bird Index, and the indicator of High Nature Value farming should be maintained and improved. Opening the indicators’ list to scientific evaluation and participation and clarifying the (currently non-transparent) process of updating the indicators would pave the way for future improvements of the indicator framework.
8. **Strengthen environmental monitoring and enforcement** to ensure that CAP instruments lead to desirable results. Annual monitoring (e.g. using the EU's reporting system to account for yearly changes in land-use/cover and management) is imperative for evaluating effectiveness and efficiency, enabling policy makers and land managers to react promptly to developments, providing incentives and placing efficient sanctions in cases of infringements of the requirements. These data must be made open and freely available for science and independent impact evaluation, within a reasonable time. To reduce complexity, financial reporting and reporting for sustainability indicators should be separated.
9. **Identify and address global impacts of the CAP especially in the global South**, to achieve a reduction of environmental leakage and global negative land-use effects as well as market distortions by EU agriculture, and to comply with the EU's principle of ‘Policy Coherence for Development’ (Article 208 of the Treaty of the European Union; EC, 2019a; Matthews 2018a, 2018b). The EU needs to strive for a better understanding of the impacts of its agricultural sector on developing countries' ability to meet the SDGs, and the roles of agricultural payments (Yang, Lupi, Zhang, Chen, & Liu, 2018) and unsustainable imports, especially of animal-derived products, feed and biofuel (Barthel et al., 2018; Matthews 2018a, 2018b; Schulmeister, 2015). Beyond the CAP, strengthening international agreements and environmental governance systems, as well as communicating about sustainable consumption levels that reflect European and global capacity, are options here.
10. **Improve governance of the CAP and its reform** in order to enhance transparency, accountability, participation and knowledge-uptake in line with SDG 16, and thereby regain legitimacy and public trust (Pe'er et al., 2019). This requires opening and enabling public scrutiny of data, CAP-reform negotiation documents and implementation data, throughout the policy cycle and prior to approval. Conflicts of interest in decision-making and implementation must be identified and managed, and a more inclusive participation enabled. A more proactive integration of all affected DG's in CAP policy formulation would facilitate more policy synergies and coherence.

Overall, the breadth of scientific evidence, best-practice examples, decision-support tools and sustainability assessments should be integrated more effectively into the CAP design and implementation, in a way that acknowledges and addresses the expectations of European citizens, the multi-functionality of agricultural lands, the diversity of affected stakeholders and all three dimensions of sustainability – social, economic and environmental.

## 4 The European Commission, Council and Parliament need to take Ambitious and Responsible Actions

Sustainability is a top societal priority and an urgent challenge. It is enshrined as a goal in the Treaty of the European Union (European Union, 2016). Given the documented poor performance of the CAP with respect to sustainability, business as usual is no longer an option. Urgent and efficient actions are needed to ensure environmental and social sustainability and long-term food security.

Transforming the CAP to help farmers adapt to the sustainability challenges would serve as a landmark for the new European Commission and the Green Deal, but it will require political courage to overcome a resistance to change. Despite a potential slow-down of the reform process, it is critical to reflect on the unequivocal scientific evidence behind the demands made by civil society to direct the CAP towards sustainability targets. We therefore call on the Commission, Parliament and Council to fulfil their responsibility toward current and future generations (Hagedorn et al., 2019) by ensuring a high level of environmental and climate protection, investing in healthy food and diverse landscapes and promoting rural vitality and citizens’ well-being.

The scientific community stands ready to support the process with the knowledge and tools required for transformative changes, both at the national and EU levels, as personally indicated by above 3,600 signatories to the preprint version of this manuscript. The preprint version can be found under (https://doi.org/10.5281/zenodo.3666258) (Pe'er et al., 2020a) and the full list of signatories available under (https://doi.org/10.5281/zenodo.3685632) (Pe'er et al., 2020b).

## Figures and Tables

**Table 1:** Ten action points, corresponding targets and examples of measures for their implementation. Policy targets should be S.M.A.R.T, i.e. specific, measurable, attainable, relevant and time-bound. However, here we avoid perscribing quantitative time-bound targets, because a) all issues must be addressed as soon as possible, and b) specific targets may differ among Member States (MSs). The proposed measures demonstrate that alternative paths exist by which targets could be met. Measures that may go beyond the CAP (in its current scope), namely relating to food policies and the so-called ‘Farm to Fork Strategy’, are marked with an *F2F* icon 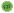

Actions	Targets	Specific measures for the EU and MSs
Transform Direct Payments into payments for public goods	The current income support (based on area alone) is fully replaced with a payment system supporting an effective provision of public goods by farmers, aligned with both environmental and socio-environmental dimensions of sustainability	Transform Direct Payments using (a combination of) the following measures: Phase-out payments, in both Pillars, with environmental damage (including activities with high use of non-renewable resources, such as fuel consumption and permanent conversion of land) Cancel coupled Direct Payments for intensive production systems as most harmful CAP subsidy with adverse environmental and social impacts (OECD, 2017) Convert decoupled Direct Payment elements into payments for environmental performance (e.g. within Eco-Schemes), and revise payments not aligned with environmental or socio-environmental goals Shift funds to the Rural Development Programmes and, within them, to Agri-Environment-Climate Measures (AECM) or other instruments benefitting the provision of public goods Reward Member States (MSs) for shifting budgets to targeted payments for public goods and/or to Pillar 2 and, within it, to AECM and similarly beneficial instruments Set co-funding requirements on Pillar 1, while reducing them from Pillar 2
Provide sufficient support for effective climate change mitigation	A reduction of GHG emissions in the agricultural sector is achieved	Revise and reduce payments for intensive animal production (starting with coupled support and continuing through decoupled Direct Payments) Expand instruments supporting a transition to sustainable animal production intensities Revise the application of the ‘Rio Markers methodology’, to register only concrete measures for GHG reduction (especially under AECM and Eco-Schemes) rather than payments with uncertain impacts on emissions Strengthen or introduce financial support for ○ rewetting of peatlands (Röder, Henseler, Liebersbach, Kreins, & Osterburg, 2015; WBAE, 2016) ○ paludicultures (i.e. wet agriculture or forestry on peatlands; Buschmann et al., 2020; GMC, 2019) ○ nature-based solutions jointly addressing climate risks and biodiversity ○ termination of conversion of organic soils to arable land (Tiemeyer et al., 2020) ○ natural-forest restoration and rewilding (Perino et al., 2019) through designated instruments (e.g. payments for forest holders) Demand MSs to prove that their support schemes lead to actual reduction in GHG emissions using full accounting or emissions' analysis Link farm risk management (i.e. insurance) to within-farm mitigation strategies (e.g. maintaining landscape features and vegetation cover to reduce drought, erosion or fire risks) to help building resilience and adaptiveness rather than compensating for losses 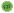 Develop and introduce EU-wide and regional labelling of farming products with low CO_2_ footprint 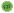 Support education and communication on healthy and balanced diets that focus on pesticide-free, regionally produced, seasonal, low CO_2_ foods and reduced reliance on animal production 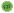 Provide incentives to reduce food-waste, transport distances (‘food miles’) and packaging
Provide sufficient support for biodiversity protection and restoration	A zero decline, followed by increasing farmland biodiversity expressed by indicator species and habitats, is achieved at the earliest possible point in time	Cancel the asymmetric budget cut on Pillar 2 proposed for the financial period of 2021–2027 Secure a significant budget for environmental measures in both pillars Restore the pre-2009 requirement for CAP recipients to set aside at least 10% of agricultural area for semi-natural habitats without production, like buffer strips, fallow land or landscape features Enlist and demand a minimum allocation to measures essential for biodiversity and associated ecosystem services: extensive arable land, buffer strips, fallow land, landscape elements (terraces, hedges, trees on farms etc.), high-diversity grasslands, wetlands, peatlands, and, at the landscape scale, High Nature Value farmland and mosaic landscapes (Lomba et al., 2020) Expand targeted budget for, and enhance farmer-profits from, implementing effective (‘dark green’), specific and/or complex AECM and Eco-Schemes for biodiversity Extend support for extensive grazing contributing to the provision of public goods Improve requirements for, and remuneration of, organic farming that complies with biodiversity-related criteria (e.g. include space for nature) as well as other sustainability criteria (Dainese et al., 2019) Improve the framework for calculating ‘cost incurred’ and ‘profit foregone’ and to generate greater benefits from public goods payments (for both biodiversity and climate) to increase effectiveness and efficiency
Support innovative approaches to design and implement measures addressing environmental challenges	Innovative agri-environment options with proven benefits are introduced across the EU and their uptake by farmers has successfully increased	Require a minimum allocation by all MSs for innovative approaches such as: ○ result-based payments (or payment components), i.e. oriented toward ecological/environmental results (Herzon et al., 2018; Schroeder, Isselstein, Chaplin, & Peel, 2013) ○ collaborative implementation or other forms of local and innovative initiatives (see also Action 5) ○ auction models for the provision of AECM where suitable (Iftekhar & Latacz-Lohmann, 2019) Implement proposed approaches to reduce administrative costs of agri-environmental support without compromising targets (WBAE, 2019b) Explore means to improve farmers' motivation and participation in AECM, for example through: ○ higher flexibility and adaptability and more participatory approaches ○ enhancing financial and knowledge support for local initiatives through existing instruments (such as support for small farmers/investments) and initiatives (such as the European Innovation Partnership [EIP] and the evolving Community-Led Local Development [CLLD]) ○ using AKIS and other tools to enhance knowledge of the impacts of different farming actions on public goods, and increase the feedback to farmers and other actors
Enhance spatial planning and collaborative implementation, and the application of landscape-level measures	Spatial distribution of measures is improved to achieve higher efficiency of agri-environment payments and contribute to the EU's Green Infrastructure	Require MSs to employ spatial planning and landscape-level implementation under AECM and Eco-Schemes, and provide rewards for MSs (e.g. by reduced co-funding) and for farmers (e.g. by higher remuneration or labelling) Allocate budgets for piloting and exploring collaborative implementation approaches by farmers Link up Natura 2000 management plans with the utilization of AECM and Eco-Schemes within and beyond protected areas Encourage environmental farm management plans Employ longer-term contracts with farmers to improve both income security and ecological benefits of such efforts
Demand Member States to set S.M.A.R.T (specific, measurable, ambitious, realistic and time-bound) targets in their Strategic Plans	All Member States have defined S.M.A.R.T targets to ensure effective and efficient implementation toward fulfilling all CAP objectives	Require MSs to develop S.M.A.R.T targets in close consultation with scientists and other experts Sharpen the requirements and standards for the design and evaluation of MS's Strategic Plans, especially in terms of target-setting Revise the Performance Bonus (article 123 in EC, 2018) to ensure it incentivizes ambitious target-setting Demand MSs to clarify how they intend to address and reduce trade-offs between objectives Allow partial approval of Strategic Plans to enable approval of well-justified sections while others are revised as needed
Revise the set of indicators	EU- and MS-specific lists of indicators are based on the best available science and in accordance with SDGs, post–2020 CBD's targets and UNFCCC	Open the indicators' list to scientific evaluation and participation Clarify the currently non-transparent process of updating the indicators Redesign the set of result indicators using best knowledge so that they can support the comparison of political priorities between MSs and regions as well as for the timely monitoring and readjustment of Strategic Plans Expand the list of result indicators to ensure they balance all CAP objectives and are coherent with the SDGs Link result indicators with existing data monitored and reported by farmers (see Action point 8) to account for feasible tracing of land-use changes and supporting sustainable, adaptive farm management (mowing regime, grazing intensity, use of chemical outputs) Expand the list of impact indicators to cover all CAP objectives: reintroduce the HNV indicator (i.e. maintain it in the current list); include well-tested biodiversity indicators such as the Butterfly Grassland Indicator; include proposed indicators on farm-economy, health and well-being
Strengthen environmental monitoring and enforcement	The monitoring and management tools are adequately expanded to trace the CAP impacts and to ensure that payments lead to desirable results	Allocate a clearly defined EU budget for monitoring of CAP impacts on the environment in all MSs (see Geijzendorffer et al., 2016) Revise monitoring requirements together with scientists and relevant bodies to expand the extent and frequency of monitoring with respect to Cross Compliance, Eco-Schemes and AECM Support development, testing and implementation of emerging technologies and approaches (such as remote sensing, citizen science, DNA-based methods) for data collection and analysis across all MSs (ECA, 2020) Redesign the control and sanctioning mechanisms for putting greater emphasis on addressing potential environmental harm and less on mere ‘formal’ errors Disconnect administrative reporting requirements (=outputs/results) from performance indicators on socio-economic and environmental impacts of the CAP Make monitoring data, especially the EU's Integrated Administration and Control System (IACS) and the Land Parcel Identification System (LPIS) data, open and freely available for science and independent impact evaluation; and remove current delays in data releases to allow rapid assessment of progress against targets Implement yearly monitoring for both results and impact indicators where possible and sensible, using the EU's reporting systems (e.g. IACS, LPIS) to account for changes in land-use/cover and management Enhance law enforcement to ensure compliance with requirements, including allocating sufficient resources (staff and funding) to this within the Commission Work on synergies with existing or emerging farmland biodiversity monitoring schemes at the MS and EU level
Identify and address global impacts of the CAP especially in the global south	A measurable reduction of environmental leakage, global negative land-use effects and market distortions by EU's agriculture is achieved, complying with the EU's principle of ‘Policy Coherence for Development’ (PCD)	Abolish payments leading to adverse land-use changes and market distortions outside of the EU Develop a strategy for European agriculture with coherent CAP support to assure that agricultural production satisfies EU demands within the planetary boundaries 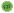 Complement certification and product standards with governmental regulation and legal arrangements in both producing and consuming countries to reduce losses of habitat and biodiversity in tropical forests and other ecosystems (Lambin et al., 2018; Milder, Newsom, Lambin, & Rueda, 2016; Tayleur et al., 2017) 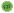 Modify trade agreements to require common standards and tying the trading in food commodities with national and EU agriculture, food and sustainability policies
Improve governance of the CAP and its reform in terms of transparency, accountability, participation and knowledge-uptake	Achieve a measurable improvement in transparency, accountability and public participation in line with SDG 16	Open negotiation and implementation documents (such as national Strategic Plans) for public evaluation prior to approval, to enable public scrutiny of data, processes and outcomes throughout the policy cycle. Documents should be made available in both national languages and in English (WBAE, 2019b) Improve political structures for consultation and public participation during the generation of national Strategic Plans as well as during CAP implementation periods in order to enhance adaptive learning for improved sustainability performance Implement a joint decision-making process for DG AGRI and DG ENVI, as well as agricultural and environmental ministers in the Council, to reflect a broader range of affected sectors and stakeholders Improve the use of modelling and scenario building in all ex-ante evaluations to assess more profoundly whether proposed changes can generate the intended improvements, while applying the precautionary principle Decouple the financial decision-making from issues regarding policy design and targeting within the CAP Enhance structured participation of scientists in CAP assessment and policy reform processes

## Box 1 The CAP in a nutshell

- As one of the founding policies of the EU, the CAP was established in the Treaty of Rome of 1957, aiming to increase productivity, enhance farmers' income, stabilize markets and ensure food supplies and reasonable consumer prices. The CAP has been subject to numerous reforms and is currently undergoing another reform process. The CAP is now mainly an agricultural funding policy, with a budget of €58.4 bn./year (as of 2019), i.e. 36% of the total EU budget (EC, 2019c). It is divided into two ‘Pillars’.
- Pillar 1 consists of income support and market measures. Income support is granted through different types of Direct Payments (€40.4 bn., 69.4% of CAP in 2019; Figure 1). Direct Payments are mostly paid per hectare and are conditional on compliance with various regulations including environmental aspects (e.g. ‘Good Environmental Agricultural Conditions’, or GEAC, in ‘Cross Compliance’). Since 2014, 30% of Direct Payments are linked to three ‘Greening’ requirements, evaluated as mostly ineffective (ECA, 2017; Pe'er, Lakner, et al., 2017). €5.7 bn. of the Direct Payments is coupled to specifictypes of production, such as sugar, cotton, beef and veal, dairy, sheep and goats and protein crops. €3.0 bn. (5.3%) is for market support and stabilization.
- Pillar 2 refers to the Rural Development Programmes (€14.4 bn.; Figure 1), and includes instruments supporting rural areas (€8.6 bn.) as well as Agri-Environment-Climate Measures (AECM), payments for organic farming and Natura 2000 sites (€3.5 bn.). The latter three are regarded as ecologically effective (Batáry et al., 2015). Support for Areas of Natural Constraints (ANC, €2.5 bn.) has mixed environmental impacts (Oppermann, Beaufoy, & Jones, 2012).
- For the CAP post-2020, the European Commission proposes (a) a new set of objectives, (b) a new delivery model, granting more flexibility to Member States (MSs), (c) a requirement to develop national Strategic Plans, delineating how MSs will set and implement targets, and (d) a new ‘Green Architecture’ with higher environmental requirements in Cross Compliance (‘enhanced conditionality’) and voluntary ‘Eco-Schemes’ replacing the obligatory but ineffective ‘Greening’ measures in Pillar 1 (Pe'er et al., 2019).
